# The Status of the Drug Business in Atlanta

**Published:** 1903-09

**Authors:** Samuel A. Visanska

**Affiliations:** Visiting Physician to the King’s Daughters Hospital, Hebrew Orphans Home, Woman’s Co-operative Home, Children’s Dispensary, City Board of Missions


### THE STATUS OF THE DRUG BUSINESS IN ATLANTA.

BY SAMUEL A. VISANSKA, PH.G., M.D.,

Visiting Physician to the King's Daughters Hospital, Hebrew Orphans Home, Woman's Co-operative Home, Children's Dispensary, City Board of Missions.

It has been some time since I have had the pleasure of reading a paper before this Association, and thought it would not be amiss to go out of our usual line and give the status of the drug business in Atlanta. Having graduated from that grand old institution, the Philadelphia College of Pharmacy, I feel that I am, in a measure, competent to criticize, and not too harshly, the drug business from both the standpoint of a physician and pharmacist. It was my good fortune to come to this great city about eight years ago, and coming as I did a perfect stranger, my first friends were among the druggists. Many of my best friends to-day are druggists, and you might then ask the question, why does the doctor read this paper? The answer from my standpoint is, duty comes before all else, friendship is a second consideration, and if the "cap fits" even my best friends, they must wear it gracefully. I shall then endeavor to point out a few of the leading horrors of the drug business, and leave it to you in your discussion to do the rest.

*Substitution.*—First and foremost comes substitution, which I consider one of the greatest crimes next to murder. A man who will substitute will steal or lie, because they steal the ingredients which you ordered and put something else in its place; they will lie, because you can not get them to acknowledge it, even if caught. Why, the druggists substitute anything from aq. destillata to Gude's Pepto-Mangan, and how easy it is at times to catch them if you have had a knowledge of the drug business. The reason I mention aq. destillata first is, the oculist often orders nitrate of silver and distilled water; unless the druggist fills the prescription as ordered you will in a short time have a cloudy mixture of chloride of silver. Some time ago I wrote a prescription containing chlorate of potash, glycerine, tr. chloride of iron, borolyptol and water; upon my return to the patient's house I asked to see the medicine, and to my surprise found an inky mixture, and upon investigation found the druggist had used listerine in place of borolyptol. Now the point about that is this, if I had wanted listerine and ink, I would never have ordered borolyptol. Another time I ordered fld. ext. hamamelis in a prescription; the druggist filled it with distilled ext. of witch-hazel. Not a few substitutions are made either through laziness or ignorance, as an example, when you order inf. digitalis, it is invariably made from the fld. ext. Few pharmacists realize that the action of the preparation will be just opposite that which is desired, because the fld. ext. is made with alcohol which extracts certain alkaloids that act as a heart stimulant; these are digitalin, digitalein and digitoxin. The alkaloid desired, digitonin, is insoluble in alcohol, but readily soluble in water, and seems to be the principle upon which the diuretic effect of the drug depends, this alkaloid antagonizes the others, acting as a depressant. The proprietary articles come in for their full share of substitution, and it might pay a few of the leading firms to employ detectives.

*Graduates.*—It might be of interest to know how your druggists are made, and who fills the prescriptions. A few days ago I called up a few of your leading druggists and inquired if they were graduates or state board licentiates; if they went behind the prescription counter, how many clerks they employed, and if they were graduates. You will be surprised to know how many drug stores are conducted where the proprietor never goes near the prescription counter. A few of the clerks are graduates of pharmacy, the majority are State boarders. I also inquired if there were any undergraduates in the stores and the answers came, no. Good heavens, are these druggists shot up out of the earth, with just enough knowledge to pass the State board, or where do they obtain their experience. There is an old saying that "musicians are born, not made"; in the drug business it is just the reverse. It seems that the day of the preceptor has passed, and it is a sad day for both physician and patient when it does. It takes more than a head full of chemical formulas, or to be able to roll a pill or fold a powder, to become the thorough druggist. You need to be taught the groundwork by some one competent to teach. You must be willing to start with washing bottles and sweeping the floor; you must acquainyourself with the color, odor and taste of the different drugs; you must watch your preceptor pack a percolator, make an infusion, roll a pill, fold a powder; you must learn accuracy and promptness, and above all to give just what the physician orders. You must also learn the business side of it until finally you drift into a full-fledged Ph.G. A college education without several years' practical experience is not worth writing your name on a diploma. I think that the State board is a farce as it has been conducted in the past. You can cram your head full of theory and with very little experience pass. They have degrees, good, better, best, and as far as I know, one has the same privileges as the other. I will give you an example of this farce: some years ago a physician from a small town wrote me, asking if I would quiz him for the State board. I replied, yes. The doctor came to me at once, and for a few days I put him through the questions usually asked at State board examinations, and this fellow passed. There are so many reasons why a druggist should be practical and have practical knowledge, that it would take all night telling you. Gentlemen, of all the places of trust, the druggist comes first. The lives of thousands are in his hands; your reputation in a measure is at stake. The druggist acts as agent of the physician, and in so doing should in every way protect his interests. The pharmacist is supposed to have even a better knowledge of drugs than the physician, because he is there to correct errors often made by the busy physician. As an example you might wish 1 gr. of strychnin in a solution, and by a slip of the pen it reads 10. The druggist in reading the prescription detects the error at a glance, and telling the patient, "It will take some time to fill the prescription," he in the meantime, communicates with the physician, the error is corrected, and the patient is none the wiser. Now it is the duty of the physician to extend thanks.

*Checking Prescriptions and Errors.*—Not a few druggists claim to have a system of checking prescriptions to avoid errors in filling prescriptions and writing the label. This is a most admirable method, but I have failed to see this system in Atlanta. It makes no difference how careful you are, or how much experience you have had, you can make errors. There are two methods, the double check, where more than one clerk is employed, and the single check system. Time will not permit me to explain these. A few days ago I wrote a prescription containing, among other things, cit. caffein and quinine; this was ordered made into capsules. I also ordered calomel powders. The next day I asked the patient how many capsules she had taken, and to my surprise she answered none, but that the powders were terribly bitter and made her vomit. I explained that I did not intend ordering powders but thought I had ordered capsules. I took the number of the prescription, and upon my return to the office called up the druggist and ask that he repeat the prescription ; he said, make into capsules. I then told him of his error and asked that he send for the powders and put them into capsules. Now this was a reflection upon me, ordering such bitter medicine, when there are so many ways of disguising the taste. A check system would have prevented this. Morphine has been put in prescriptions for quinine, laudanum for harmless tinctures, etc., etc.

*Counter prescribing.*—No doubt you all know that the druggists run us a close second when it comes to prescribing, and they have formulas to cure anything from a corn to a prolapsed anus; and who is the loser? They are, because the patient will scarcely pay them over ten or fifteen cents ; whereas, if they would refer them to a physician, they would get one or two prescriptions which would bring from twenty-five cents to a dollar. Besides, it is illegal, and steps should be taken to prosecute such.

*Refilling Prescriptions.*—One of the greatest evils that exist today is the refilling over and over again a prescription which the patient, his friends, relatives and acquaintances bring to you. This habit should and can be stopped if the physicians of Atlanta want it done. It is not allowed in some cities, and should not be tolerated here.

*Delivery System.*—Until recently the delivery of prescriptions to the patient has been quite unsatisfactory ; often an order left in the morning would not be delivered until late in the afternoon. Thanks to one or two enterprising firms this serious obstacle has been overcome.

In closing must say that we should not be unmindful of the fact that we have a few druggists whose characters are above reproach, who, I am sure, would not tolerate the evils mentioned. Let us, then, seek those whom we can trust, and give to them our moral and business support, and in the meantime find out the others, who will, in a short while, bury themselves.

*405-406-407 Peters Building.*

